# Evaluation of inflammatory vascular responses in patients with severe periodontitis by contrast-enhanced perfusion dental MRI

**DOI:** 10.1186/s41747-025-00634-6

**Published:** 2025-09-24

**Authors:** Arne Lauer, Artid Skenderi, Luisa Schulte, Alexander Juerchott, Meysam Sohani, Maurice Ruetters, Franz Sebastian Schwindling, Peter Rammelsberg, Mathias Nittka, Sabine Heiland, Martin Bendszus, Tim Hilgenfeld

**Affiliations:** 1 Department of Neuroradiology, Heidelberg University Hospital, Heidelberg, Germany; 2 Private Practice, Schwalmtal, Germany; 3 Section of Periodontology, Department of Operative Dentistry, Heidelberg University Hospital, Heidelberg, Germany; 4 Department for Prosthetic Dentistry, Medical University Innsbruck, Innsbruck, Austria; 5 Department of Prosthodontics, Heidelberg University Hospital, Heidelberg, Germany; 6 Magnetic Resonance, Siemens Healthineers AG, Erlangen, Germany

**Keywords:** Alveolar bone loss, Cone-beam computed tomography, Magnetic resonance imaging, Perfusion, Periodontitis

## Abstract

**Background:**

Periodontitis is characterized by the inflammatory destruction of tooth-supporting alveolar bone. Dental magnetic resonance imaging (MRI) using dynamic contrast-enhanced perfusion can potentially detect vascular inflammatory responses. This study aims to assess the feasibility of perfusion dental MRI and characterize periodontal lesions with perfusion profiles.

**Materials and methods:**

In this prospective study, 19 patients with severe periodontitis underwent pretreatment 3-T dental MRI with T2-weighted, high-resolution dynamic contrast-enhanced T1-weighted perfusion protocol, and contrast-enhanced T1-weighted fat-suppressed sequences as well as cone-beam computed tomography (CBCT). Periodontal bone lesions were segmented semiautomatically using a multistep threshold-based algorithm, guided by T1-weighted contrast enhancement, T2-weighted hyperintensity, as well as CBCT-based bone loss. Volumetric analyses and clinical data were compared with perfusion parameters.

**Results:**

In all 95 assessed periodontal lesions, perfusion parameter elevations were significantly different when compared to normal distant bone (*p* < 0.001 to 0.026). Moreover, structurally normal-appearing bone adjacent to T2-hyperintense/T1-contrast-enhancing signal alterations exhibited increased permeability (*p* = 0.036–006) but showed no significant change in blood flow (*p* = 0.270) compared to bone control areas. Lesions with bleeding showed higher vascular permeability and blood flow markers than lesions without bleeding (*p* = 0.004–0.006). Additionally, lesions with excessive edema and areas of bone loss exhibited significantly elevated permeability and blood flow parameters (*p* = 0.001–0.028).

**Conclusion:**

Perfusion dental MRI for periodontal lesion assessment is feasible. Permeability/perfusion parameters elevations are related to clinical signs of inflammation and CBCT-based bone loss, with the potential for detecting early inflammatory responses.

**Relevance statement:**

Perfusion dental MRI effectively characterizes periodontal disease by detecting inflammation-related vascular changes beyond structural imaging on CBCT and conventional MR, offering potential for improved diagnosis, monitoring, and treatment evaluation. Longitudinal studies are needed.

**Key Points:**

- Perfusion dental MRI detects increased blood flow and vascular permeability in periodontal lesions.
- Increased permeability in adjacent bone suggests early inflammatory changes before structural loss.
- Dental MRI perfusion metrics could aid early lesion detection and monitoring of periodontitis.

**Graphical Abstract:**

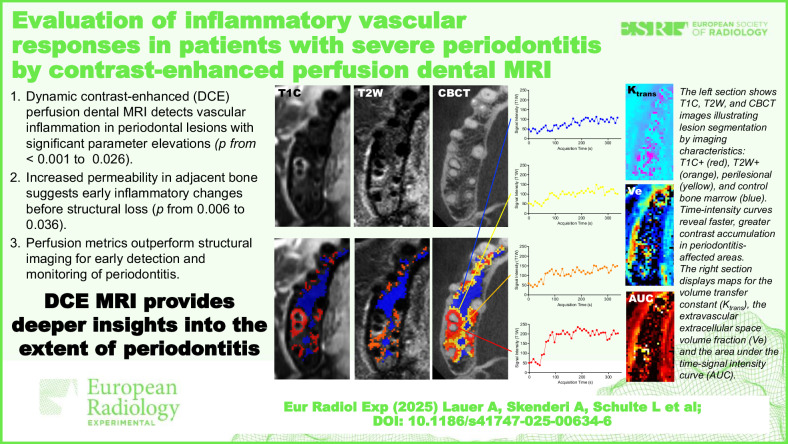

## Background

Periodontitis is a chronic inflammatory condition that progressively destroys periodontal structures and adjacent bone. It has a high prevalence, affecting approximately 11% of adults globally, and significantly impacts quality of life [1, 2]. Additionally, periodontitis is linked to the worsening of various systemic disorders, such as cardiovascular disease, diabetes, and cancer, while proper treatment of periodontitis has been shown to improve morbidity and mortality outcomes for these non-oral diseases [3].

Despite its importance, clinical decision-making mainly relies only on orthopantomography and clinical examination [4]. Clinical examination results, however, perform poorly in predicting disease progression [5]. While clinical indicators like bleeding on probing are commonly associated with active inflammation, these assessments are limited to areas where attachment loss has already occurred. Moreover, a previous study revealed that only 30% of sites presenting with bleeding on probing showed disease progression measured by clinical attachment loss [6]. Two-dimensional and three-dimensional x-ray imaging techniques only depict loss of bone. Additional information, such as bone at risk, is not provided, even though this may be beneficial for guiding clinical decision-making and enhancing treatment response [7]. Recently, dental magnetic resonance imaging (MRI) has been shown to identify intraosseous edema extending beyond areas of bone loss in patients with generalized periodontitis, providing additional information compared to orthopantomography [8, 9]. Excessive edema can be characterized by a mismatch between lesion volumes delineated on T2-weighted and contrast-enhanced T1-weighted maps, potentially reflecting various stages and aspects of inflammation, such as hyperemia, interstitial edema, cellular infiltration, medullary necrosis, bleeding, and fibrovascular ingrowth [10, 11].

Stratifying lesional properties through dental MRI could provide valuable insights for monitoring periodontitis therapy and targets for early intervention [12]. During active inflammation, the release of inflammatory mediators from granular cells is expected to alter cytokine-driven vascular responses, which could further influence imaging characteristics [13]. Dynamic contrast-enhanced (DCE) MRI uses gadolinium-based contrast agents to monitor temporal changes in signal intensity by capturing sequential images before and after contrast injection. As the agent enters and exits the tissue, it alters signal intensity temporarily, with these variations analyzed to derive physiopathological parameters related to blood flow, vessel permeability, and tissue volumes for each voxel. The area under the time-signal intensity curve (AUC) reflects contrast agent accumulation and washout, with higher values indicating increased blood flow. The volume transfer constant (K_trans_) and the extravascular extracellular space volume fraction (V*e*) provide insights into contrast movement and vascular permeability.

Evaluating contrast kinetics thereby may effectively measure vascular responses, making it a valuable tool for detecting inflammatory hyperemia and vasogenic edema [14]. This approach provides additional insights into lesions beyond what is available from structural imaging alone. Moreover, in cases of bone destruction, the granulating tissue that replaces bone is expected to show differences in vascular density and tracer kinetics compared to intact bone, offering further differentiation of tissue pathology.

This study aims to: (1) evaluate the feasibility of DCE MRI perfusion imaging in patients with periodontal disease; (2) characterize the perfusion profiles of periodontal tissues using DCE MRI based on tissue segmentation derived from structural imaging parameters; and (3) assess correlations of these parameters to clinical and imaging-based markers of disease activity.

## Materials and methods

### Study participants

This study received approval from the University of Heidelberg’s local research ethics committee (S-452/2010, date of approval: 6/23/2020). All research was performed in accordance with local guidelines and regulations and in accordance with the Declaration of Helsinki. All participants provided written informed consent.

For this cross-sectional study, subjects were drawn from an ongoing cohort study of periodontitis and selected based on the presence of advanced periodontitis (stage III, grade B/C or higher) [15] and available high-resolution three-dimensional (3D) T2-weighted and T1-weighted structural sequences, DCE MRI perfusion, and CBCT, enrolled between September 2017 and December 2022. Only patients who were untreated for periodontitis for at least 3 months before the clinical and radiological examinations were included. Exclusions encompassed contraindications to 3-T MRI or MRI contrast agents, age under 18 years of age, pregnancy, or claustrophobia. Data from incomplete or motion-compromised magnetic resonance perfusion were also excluded. The indication for CBCT, performed the same day as MRI, aimed to assess severe maxillary molar furcation involvements, potential osteomyelitis, and surgical implant planning.

### Clinical examinations

Clinical examinations were conducted by two experienced dentists (M.S. and F.S.S.) who were calibrated before the study commenced. Probing pocket depths and probing attachment levels were measured to the nearest millimeter at six sites (mesiobuccal, buccal, distobuccal, distopalatal, palatal, and mesiopalatal) using a periodontal probe (HS-Parodontometer Figur CP15; Henry Schein Inc.). Bleeding on probing (BOP) was recorded at each of the six probing sites following a single probing. A tooth was considered positive for BOP if bleeding occurred at any one or more of the adjacent probing sites. In addition, the BOP percentage was calculated based on the number of all bleeding sites upon probing divided by the total number of sites probed.

Periodontitis was diagnosed according to the 2017 “World Workshop on the Classification of Periodontal and Peri-Implant Diseases and Conditions” [15]. Therefore, individual teeth were classified as affected if clinical attachment loss of ≥ 1 mm, combined with probing depths of ≥ 4 mm, was present at any site.

### MRI

MRI was performed on a 3-T system (MAGNETOM Trio, Siemens Healthineers) with a 15-channel specialized dental coil (Mandibula, Noras MRI products GmbH).

Table 1 summarizes technical parameters of the following sequences:

1. A 3D fat-saturated T2-weighted “multislab acquisition with view-angle tilting gradient”—MSVAT was performed, based on a “sampling perfection with application-optimized contrasts using different flip-angle evolution”—SPACE research sequence;
2. Baseline T1-weighted 3D gradient-echo sequence “volumetric interpolated breath-hold examination” (VIBE) for T1-mapping;
3. Dynamic T1-weighted VIBE sequence as in point 2, before/after gadolinium-based contrast (0.1 mmol/kg gadoterate meglumine, Dotarem®, Guerbet) injection at 5 mL/s, succeeded by a 25-mL saline flush, over 42 cycles;
4. Single final contrast-enhanced 3D T1-weighted isotropic (VIBE) sequence with Dixon-based fat suppression.

**Table 1:** Magnetic resonance imaging sequence parameters and order of acquisition

Sequence order, weighting and name	(1) T2-weighted MSVAT-SPACE	(2) T1-weighted VIBE	(3) Dynamic T1-weighted VIBE	(4) T1-weighted VIBE with Dixon fat suppression
Scope	Structural evaluation (edema)	Baseline correction for DCE perfusion	DCE perfusion	Contrast-enhanced structural evaluation
Voxel size (mm^3^)	0.6 × 0.6 × 0.6	0.9 × 0.9 × 0.9	0.9 × 0.9 × 0.9	0.7 × 0.7 × 0.7
Echo time (ms)	236	2.46	2.46	2.45
Repetition time (ms)	2,500	4.47	4.47	15.6
Inversion time (ms)	200	–	–	–
Flip angle (°)	120	5, 8, 11, 14, 17	15	14.9
Field of view (mm^2^)	191 × 151	180 × 180	180 × 180	223 × 153
Receiver bandwidth (Hz/pixel)	521	501	501	601
Acquisition matrix	320 × 252	320 × 252	320 × 252	220 × 320
Number of sections	104	24	24	80
Acquisition time (s)	425	527	509	362

*DCE* Dynamic contrast-enhanced, *MSVAT* Multislab acquisition with view-angle tilting gradient, *SPACE* Sampling perfection with application-optimized contrasts using different flip-angle evolution, *VIBE* Volumetric interpolated breath-hold examination

The selection of the upper *versus* the lower jaw was based on the severity of furcation involvement determined from the structural sequences.

### CBCT imaging

CBCT images were obtained using a 3D Accuitomo 170 system (J. Morita Manufacturing Corp.) with settings adjusted for cylindrical volumes ranging from 4 × 4 cm to 8 × 8 cm. The device operated at a tube voltage of 90 kV and a tube current of 5 mA, completing a full 360° rotation in a total scan time of 17 s. The images featured an isotropic voxel size of 0.16 mm. To enhance accuracy and reduce patient movement, heads were stabilized using both head and chin supports during scans.

### Data processing and analysis

Digital Imaging and Communications in Medicine (DICOM) files from the study were anonymized and randomized. Retrospective motion correction by rigid alignment was applied to the dynamic raw perfusion data before analysis with the ‘T1Mapping’ and ‘PkModeling’ modules in 3D Slicer software (version 4.11.20210226, [www.slicer.org]). DCE MRI leverages contrast kinetics to detect temporal changes in signal intensity by acquiring sequential images before and after contrast agent injection [14]. Population-based arterial input functions were used to generate the following parameter maps with the method by Parker et al [16]:

1. The AUC for the first 90 s of DCE acquisition, a semiquantitative index of tissue perfusion;
2. The fractional volume of the extracellular extravascular space (V*e*), representing the proportion of tissue perfused by interstitial fluid and hence, may correlate to interstitial edema;
3. The volume transfer constant (K_trans_), which, based on the signal intensity curves, models the rate at which contrast agent moves from blood plasma to the extravascular extracellular space and is thought to reflect the combined effects of vascular permeability and capillary plasma flow.

### Image registration and analysis

**Fig. 1:**
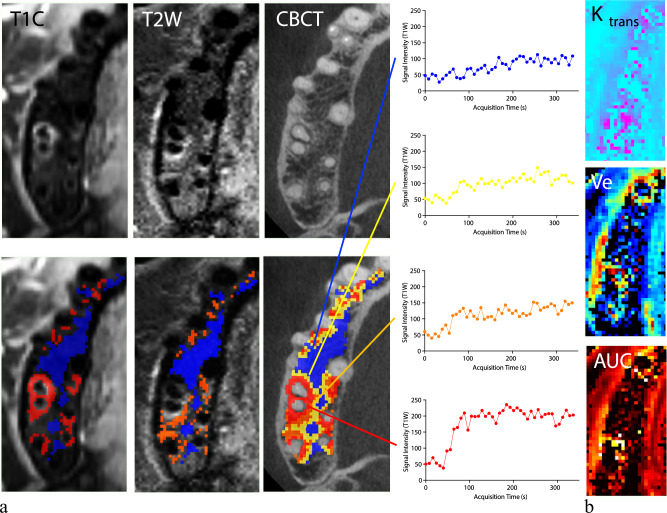
Dynamic contrast-enhanced perfusion imaging analysis. **a** T1-weighted contrast-enhanced images (T1C), T2-weighted images (T2W), and cone-beam computed tomography (CBCT) images to demonstrate the segmentation of lesions based on distinct imaging characteristics. The segmentation differentiates between periodontal lesions showing increased signals in T1C imaging (T1C+, highlighted in red), areas being hyperintense on T2W but not enhancing on T1C (T2W+, marked in orange), perilesional areas appearing normal in both T1C and T2W (PL, yellow), and the normal bone marrow distant from the lesions used as control (C, indicated in blue). The corresponding time-intensity curves depict a faster and greater uptake of the contrast agent in areas impacted by periodontitis relative to the distant bone marrow that appears normal. **b** Corresponding maps for the volume transfer constant (K_trans_), the extravascular extracellular space volume fraction (V*e*) and the area under the time-signal intensity curve (AUC)

Image datasets were converted to the Neuroimaging Informatics Technology Initiative (NIfTI-1) format and co-registered using 3D Slicer’s landmark registration tool, aligning CBCT acquisitions with T2-weighted sequences and unenhanced T1-weighted sequences to the initial dynamic T1-weighted sequence. Subsequent manual outlining of the visible lesion was performed using the segmentation tools in 3D Slicer (Fig. 1).

**Fig. 2:**
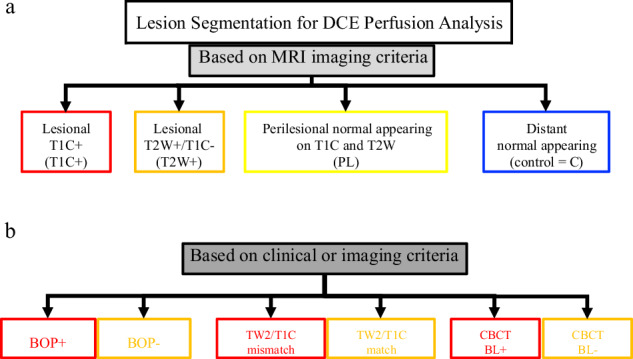
Lesion segmentation workflow. **a** First, bone lesions were segmented as being contrast-enhancing on T1-weighted images (T1C+, red), showing increased signal on T2-weighted imaging without contrast enhancement (T2W+, orange), perilesional (PL, yellow) and distant normal-appearing control bone (C, blue). **b** T1C+ regions were then dichotomized based on clinical bleeding on probing (BOP, positive *versus* negative), lesions with significant T2W/T1C mismatch (yes, *versus* no) and bone loss seen in CBCT (BL+ *versus* BL-). Within these segmentations, the average corresponding regional output for the perfusion-based maps was extracted

Initially, lesions were segmented into volumes of interest (VOIs): T1C+ (enhancing on T1-weighted and hyperintense on T2-weighted images) and T2W+ (non-enhancing on T1-weighted but hyperintense on T2-weighted images) using a semiautomated thresholding process based on intensity values exceeding those of normal bone marrow. Perilesional VOIs were delineated to include regions immediately adjacent to the lesion that showed neither enhancement on T1-weighted contrast-enhanced images nor hyperintensity on T2W sequences and was defined by expanding the outer boundary of the combined T1C+ and T2W+ VOIs isotropically by 2 mm, excluding any overlapping lesional tissue. Voxels within this expanded region were included in the perilesional VOI only if their intensity values on both imaging sequences matched the characteristics of normal bone marrow, as determined from the control VOI of distant normal-appearing bone (Fig. 2).

To further characterize the perfusion profiles of T1C+ periodontal lesions, VOIs were selected based on the following imaging characteristics and clinical features:

1. Bleeding on probing (BOP), *i.e*., VOIs were placed within T1C+ lesions on the side positive for BOP *versus* sides negative for BOP, based on clinical examination;
2. T1C+/T2W+ volume mismatch, *i.e*., segmented T1C+ lesions of teeth with mismatched T2W+ lesion volumes (exceeding the 95% confidence interval for T1C+ lesion volumes) compared to T1C+ lesions without mismatched T2W volumes;
3. Presence or absence of bone loss, *i.e*., VOIs manually placed on T1C+ lesions in areas with bone loss *versus* T1C+ lesions with preserved bone structure, as seen on CBCT.

For points 1 and 3, a VOI volume of 40 voxels was used for placement. This is based on the average lesion volume per tooth and its separation into the 6 clinical probing side (37.3 voxels). For point 2, the previously outlined volume was segmented into the two groups and used to extract mean output from the corresponding perfusion maps (Fig. 2).

Two independent readers, blinded to clinical and perfusion data, performed the VOIs placement.

### Statistical analysis

Reliability of lesion identification and placement of VOIs was assessed using the intraclass correlation coefficient and interpreted as described by Shrout [17]. Data normality was verified using the Shapiro–Wilk test. Paired *t*-tests were utilized to compare the number of clinically diagnosed teeth with periodontitis and the number of teeth with bleeding on probing and the Mann–Whitney test for probing depth and bone volume loss in CBCT between the jaw selected for perfusion analysis and the opposing jaw. Perfusion map outputs and structural MRI signal intensities were evaluated across VOIs using the Friedman test with Dunn’s corrections. One-way ANOVA with repeated measures and Dunnett’s test were applied to CBCT data, respectively.

### Use of generative artificial intelligence software

A large language model (GPT 4o, Open AI Inc.) was used for language refinement of the introduction and discussion sections of this manuscript.

## Results

Table 2 summarizes the characteristics of the 19 evaluated patients, including 4 with lesions in the lower jaw and 15 in the upper jaw. On average, each patient had 5 teeth affected by periodontitis (median, interquartile range 3–7) that were examined using MRI perfusion. Altogether, 95 periodontal lesions were assessed with all imaging modalities.

**Table 2:** Baseline characteristics of periodontitis patients (*n* = 19)

Age, years (median, IQR)	53.38 (47.37–60.12)		
Female sex, *n* (%)	10 (53)		
Smoking history, *n* (%)	3 (16)		
Diabetes mellitus, *n* (%)	4 (21)		
	**All lesions**	**Lesions within the perfusion FOV**	***p*-value**
Clinical periodontal lesions, *n* (IQR)	11 (9–16)	5 (3–7)	0.412
Clinical attachment loss, mm (median, IQR)	6 (5–8)	7 (5–9)	0.254
Max. probing depth, mm (median, IQR)	5 (4–6)	5 (4–7)	0.233
No bleeding sides (median, IQR)	12 (7–16)	7 (3–8)	0.708
CBCT lesion volume, mL (median, IQR)	0.045 (0.022–0.092)	0.065 (0.034–0.139)	0.001

*p*-values represent comparison of lesion within the perfusion FOV *versus* those outside the FOV

*CBCT* Cone-beam computed tomography, *FOV* Field of view

The delineation of lesions demonstrated good inter-reader reproducibility. The best to worst inter-rater intraclass correlation coefficient ranged from 0.817 (95% confidence interval 0.711–0.884) for the T1C+ VOI to 0.743 (95% confidence interval 0.595–0.837) for the T2W+ VOI.

**Fig. 3:**
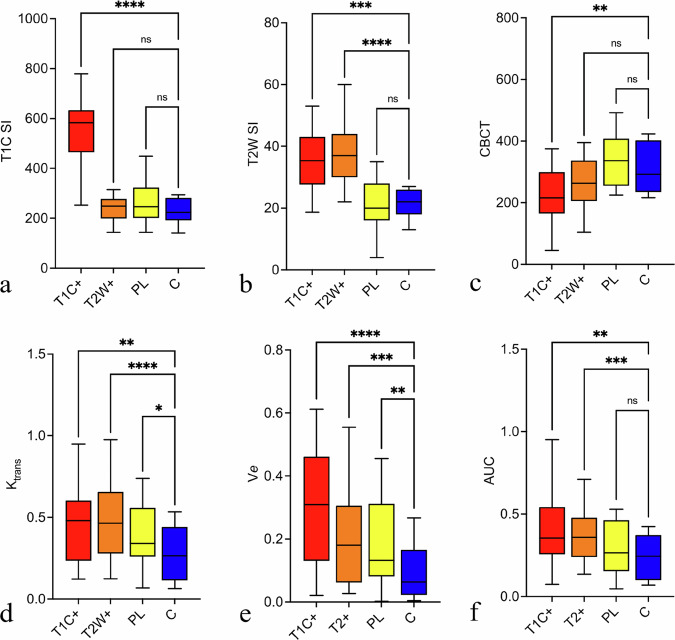
Structural imaging *versus* perfusion abnormalities in periodontitis. Tukey plots for 19 patients with severe periodontitis display average regional output from segmented areas as contrast-enhancing on T1-weighted images (T1C+, red), showing increased signal on T2-weighted imaging without contrast enhancement (T2W+, orange), alongside perilesional (PL, yellow) and normal distant control bone (C, blue) regions. The top row (**a**–**c**) shows signal intensities from contrast-enhanced T1-weighted and T2-weighted images and radiolucency from CBCT imaging. The bottom row (**d**–**f**) presents perfusion metrics: volume transfer constant (K_trans_), extravascular extracellular space volume fraction (V*e*), and area under the time-signal intensity curve (AUC), indicating vascular permeability and blood flow. Analysis performed using repeated measure ANOVA and Dunn’s *post hoc* correction. Significance levels marked as * *p* < 0.05, ** *p* < 0.01, *** *p* < 0.001

Typical periodontal lesion characteristics included T2-weighted hyperintense and contrast-enhanced areas in the periodontal space and adjacent bone. The T2-weighted hyperintensities extended beyond the contrast-enhanced areas to varying degrees (Fig. 1). While only T1C+ differed from the control region in extracted T1-weighted signal, the corresponding T2-weighted signals from both VOIs (T1C+ and T2W+) were significantly elevated compared to the control region (Friedman’s tests *p* = 0.001 and *p* < 0.001; *post hoc* Dunn’s corrections for T1-weighted imaging: T1C+ *versus* controls: *p* < 0.001; Fig. 3a; for T2-weighted imaging: T1C+ *versus* controls: *p* = 0.001, T2W+ *versus* controls: *p* < 0.001; Fig. 3b). However, no differences were observed in both T1-weighted and T2-weighted sequences between the normal-appearing perilesional zone and the more distant control region (*post hoc* Dunn’s corrections: Perilesional *versus* control regions, *p* = 0.998 or 0.999, respectively; Fig. 3a, b).

The VOI representing the T1C+ lesions exhibited lower CBCT density values relative to the control region (repeated measures ANOVA *p* = 0.001; *post hoc* Dunnett’s comparisons: T1C+ *versus* controls: *p* = 0.001; Fig. 3c). In contrast, no significant differences in CBCT density values were observed in the T2W+ VOI (*post hoc* Dunnett’s comparisons for CBCT: T2W+ *versus* controls: *p* = 0.060; Fig. 3c) or in the perilesional areas that appeared normal on both T2-weighted and contrast-enhanced T1-weighted MRI (*post hoc* Dunnett’s: Perilesional regions *versus* control regions, *p* = 0.235; Fig. 3c).

MRI perfusion of areas affected by periodontitis revealed a rapid initial uptake, followed by a plateau phase (Fig. 1, red compartment), whereas in the control regions, a gradual increase in signal intensity was observed over time (Fig. 1, blue compartment). Spatially, a radial reduction in perfusion abnormalities was observed from the periodontal space toward the periphery in both vascular permeability (K_trans_ and V*e*) and blood flow-sensitive maps (Fig. 3). Similar to structural imaging, K_trans_, V*e*, and AUC showed significant differences between T1C+ and T2W+ VOIs compared to the control VOIs (K_trans_ / V*e* / AUC for T1C+ *versus* control regions: *p* = 0.026 / = 0.001 / 0.005; T2W+ *versus* control regions: *p* = 0.001 / 0.001 / 0.006). In contrast to structural imaging, K_trans_ and V*e* (but not AUC) demonstrated significant differences between the perilesional and control regions (K_trans_ / V*e* / AUC perilesional *versus* control regions: *p* = 0.036 / 0.006 / 0.270).

**Fig. 4:**
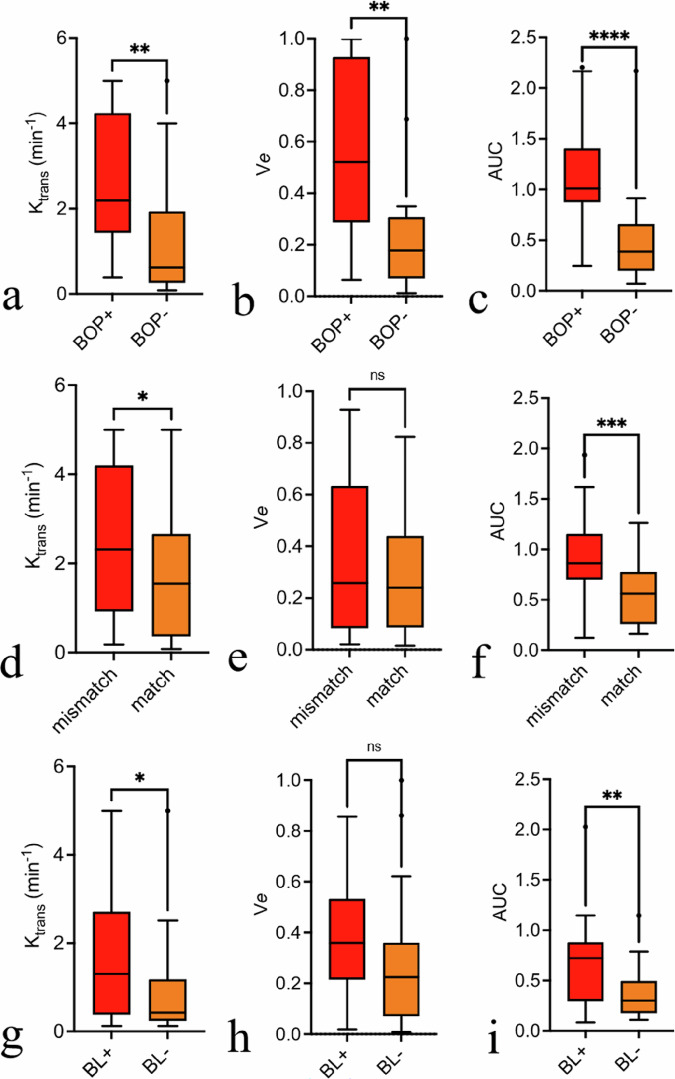
Regional perfusion characteristics in periodontitis. Tukey plots for 19 patients with severe periodontitis display average regional output for the volume transfer constant (K_trans_), extravascular extracellular space volume fraction (V*e*), and area under the time-signal intensity curve (AUC) of corresponding segmented areas based on clinical bleeding on probing (BOP, positive or negative, top row, **a**–**c**), lesions with significant T2W/T1C mismatch (yes or no, middle row, **d**–**f**) and bone loss seen in CBCT (yes *versus* no, lower row, **g**–**i**). Analysis performed using paired, two-tailed *t*-tests. T2W, T2-weighted images; T1C, Contrast-enhanced T1-weighted images. Significance levels marked as * *p* < 0.05, ** *p* < 0.01, *** *p* < 0.001

Figure 4 presents the results of dichotomizing periodontal lesions based on clinical and imaging-based characteristics. Grouping periodontal VOIs based on clinical evaluation of BOP showed that lesions in BOP-positive teeth had significantly higher K_trans_, V*e* and AUC values compared to BOP-negative teeth (K_trans_: 2.7 ± 1.56 min^−^¹ *versus* 1.27 ± 1.39 min^−^¹, *p* = 0.006; V*e*: 0.599 ± 0.468 *versus* 0.230 ± 0.245, *p* = 0.004, AUC: 0.688 ± 0.443 *versus* 0.372 ± 0.254, *p* = 0.006).

Periodontal lesions with significant T2W mismatch volumes exhibited higher K_trans_/AUC values compared to lesions without mismatch (2.486 ± 1.791 min^−^¹ *versus* 1.750 ± 1.541 min^−^¹, *p* = 0.028 and 0.932 ± 0.447 *versus* 0.575 ± 0.311, *p* = 0.001, respectively). Similarly, K_trans_/AUC values differed between areas with *versus* areas without bone loss on CBCT (K_trans_: 1.580 ± 1.434 min^−^¹ *versus* 0.943 ± 1.172 min^−^¹, *p* = 0.026; AUC: 0.688 ± 0.443 *versus* 0.372 ± 0.254, *p* = 0.006). For both comparisons, the differences in V*e* were not significant (mismatch *versus* match: 0.348 ± 0.301 *versus* 0.298 ± 0.251, *p* = 0.473 and bone loss present *versus* bone loss absent: 0.373 ± 0.252 *versus* 0.282 ± 0.2764, *p* = 0.148).

## Discussion

In this study, we demonstrated the feasibility of DCE perfusion MRI to characterize periodontal disease *in vivo*. Vascular permeability and perfusion parameters elevations were related to clinical signs of inflammation and CBCT-based bone loss. Furthermore, we evaluated the spatial extent of signal alterations associated with periodontal disease. DCE perfusion MRI, as the most sensitive imaging modality, outperformed T1-weighted imaging, fat-suppressed T2-weighted imaging, and CBCT in detecting inflammation-related abnormalities in periodontal bone tissue. These findings underscore the clinical potential of DCE perfusion MRI for early disease recognition as well as disease monitoring to improve treatment outcomes.

Periodontitis leads to inflammation-driven bone loss, characterized by osteoclastic activity and degradation of osseous tissue [7, 18, 19]. This inflammation increases water content and vascular permeability, resulting in edema, leukocyte infiltration, and protein-rich fluid accumulation, thereby enhancing T2-weighted signal intensity [10]. However, imaging signals on T2-weighted and contrast-enhanced T1-weighted sequences can also be influenced by healing-related vascular changes, making it difficult to distinguish active inflammation from reparative processes [11]. DCE perfusion MRI leverages contrast kinetics to detect temporal changes in signal intensity by acquiring sequential images before and after contrast agent injection. These signal variations are analyzed to derive physiological parameters such as blood flow, vascular permeability, and tissue volume fractions for each voxel. The area under the curve (AUC) reflects washin and washout of the contrast agent, with higher values indicating increased blood flow. Parameters such as K_trans_ and V*e* provide insights into vascular permeability, making DCE perfusion MRI a valuable tool for assessing inflammation-related vascular responses [14]. Additionally, segmentation based on structural imaging characteristics allows for the identification of distinct perfusion profiles.

The most significant deviations from the control regions were observed in the contrast-enhancing segment (T1C+), which also predominantly exhibited a loss of bony tissue, as indicated by significantly increased radiolucency in CBCT. Additionally, our findings suggest that vascular responses occur not only in areas with increased tissue water content, as detected by T2-weighted imaging, but also in the normal-appearing bone surrounding the lesion, implicating these regions in the inflammatory process. In these areas, perfusion parameters indicative of increased vascular permeability (V*e* and K_trans_) showed significant differences compared to the control region, while the parameter most reflective of blood flow (AUC) was not significantly elevated. In addition, dichotomization of lesions with excessive edema, as seen in mismatch lesions, was associated with elevated perfusion parameters compared to match lesions. This further indicates increased inflammatory activity in such lesions.

Assuming that inflammation spreads radially from the dens root, these findings suggest that heightened vascular permeability, driven by the inflammatory microenvironment, could represent one of the earliest detectable signs using multimodal MRI. Conversely, increased perfusion metrics in areas of more severe damage may reflect granulation tissue formation and associated angiogenesis, which are components of the healing process [20]. Longitudinal studies are necessary to determine the prognostic value of these parameters. Nevertheless, DCE perfusion MRI holds promise for monitoring disease progression and evaluating therapeutic interventions.

We acknowledge several limitations of our study. First, the lack of longitudinal data limits the interpretation of our findings. While we directly compared our imaging results to the clinical reference standard, *i.e*., CBCT, the predictive value of MRI can only be assessed through longitudinal studies. Although the spatial segmentation of lesions (enhancing *versus* non-enhancing *versus* normal-appearing on structural imaging) suggests that inflammatory responses may be detected using DCE perfusion MRI before structural tissue injury becomes apparent, confirmation of this potential requires follow-up data over time. In the present study, we employed a dedicated dental MRI protocol that achieved high spatial isotropic resolutions of 0.6–0.7 mm for structural imaging and 0.9 mm for DCE perfusion MRI imaging. However, the voxel size potentially averages signals from hundreds of vessels, providing only regional averages of vascular responses. Inflammatory responses are complex and likely involve multiple overlapping processes, such as hyperemia and capillary leakage, which may affect microvascular tissue perfusion and include changes below the resolution of MRI perfusion techniques.

We chose a population-based approach of arterial input functions to generate the parameter maps in order to reduce operator-induced bias and for its described superior reproducibility [16]. However, it should be noted that a population arterial input function can introduce a bias related to artifactual changes in kinetic modeling parameters if systemic circulatory factors are altered. Some of this potential bias is addressed by using statistical testing for repeated measurements. Future studies are needed to explore reliability if longitudinal data of the same patients were to be compared. In addition, the extended echo time of 236 ms chosen in the T2-weighted sequence is longer than conventional T2-weighted values. While this may improve the contrast of tissue edema, this has to date not been systematically validated in the periodontal bone. A final limitation lies in the patient cohort, which consisted exclusively of individuals with severe periodontitis. In particular, CBCT was performed based on clinical indications such as severe maxillary molar furcation involvement, suspected osteomyelitis, or surgical implant planning. This selective inclusion may have introduced a bias, further limiting the generalizability of our findings to broader periodontal populations with less advanced disease.

Despite these limitations, the apparent differences observed in segmented lesions highlight the potential of multimodal MRI to enhance our understanding of the pathophysiology of periodontal disease. This approach could offer a more comprehensive diagnostic tool, extending beyond the capabilities of traditional imaging techniques.

In conclusion, this study has demonstrated the feasibility of using DCE MRI for perfusion imaging in periodontal disease and revealed that DCE anomalies are associated with clinical signs of inflammation (K_trans_, V*e*, and AUC) and CBCT-based bone loss (K_trans_/AUC). Moreover, elevations in permeability (K_trans_ and V*e*), but not blood flow (AUC), extended beyond structural changes and bone loss detected by structural MRI and CBCT. These *in vivo* findings enhance our understanding of periodontal disease pathophysiology, suggesting that DCE perfusion MRI can provide deeper insights into the extent of periodontitis lesions and potentially serve as a marker for early disease recognition and treatment response. However, the lack of longitudinal data warrants further research. Future studies should investigate the predictive value of MRI perfusion imaging in assessing the progression or resolution of periodontal disease.

## Data Availability

The datasets generated and/or analyzed during the current study are not publicly available due to privacy laws, but are available from the corresponding author on reasonable request.

## Abbreviations

3D: Three-dimensional
AUC: Area under the (time-signal intensity) curve
BOP: Bleeding on probing
CBCT: Cone-beam computed tomography
DCE: Dynamic contrast-enhanced
K_trans_: Volume transfer constant
MRI: Magnetic resonance imaging
T1C+: Contrast-enhancing on T1-weighted and hyperintense on T2-weighted images
T2W+: Non-enhancing on T1-weighted but hyperintense on T2-weighted images
V*e*: Fractional volume of the extravascular extracellular space
VOI: Volume of interest
